# Photo-activatable Cre recombinase regulates gene expression *in vivo*

**DOI:** 10.1038/srep13627

**Published:** 2015-09-09

**Authors:** Suzanne E. Schindler, Jordan G. McCall, Ping Yan, Krzystof L. Hyrc, Mingjie Li, Chandra L. Tucker, Jin-Moo Lee, Michael R. Bruchas, Marc I. Diamond

**Affiliations:** 1 Department of Neurology and the Hope Center for Neurological Disorders, St. Louis, MO; 2 Department of Anesthesiology and the Washington University Pain Center, St. Louis, MO; 3 Department of Anatomy and Neurobiology, St. Louis, MO; 4 Division of Biological and Biomedical Sciences, Washington University School of Medicine, St. Louis, MO; 5 Department of Pharmacology, University of Colorado School of Medicine, Aurora, CO; 6 Center for Alzheimer’s and Neurodegenerative Diseases, University of Texas Southwestern Medical Center, Dallas, TX.

## Abstract

Techniques allowing precise spatial and temporal control of gene expression in the brain are needed. Herein we describe optogenetic approaches using a photo-activatable Cre recombinase (PA-Cre) to stably modify gene expression in the mouse brain. Blue light illumination for 12 hours via optical fibers activated PA-Cre in the hippocampus, a deep brain structure. Two-photon illumination through a thinned skull window for 100 minutes activated PA-Cre within a sub-millimeter region of cortex. Light activation of PA-Cre may allow permanent gene modification with improved spatiotemporal precision compared to standard methods.

Neuroscientists have generated exquisitely detailed atlases of gene expression and connectivity in the mouse brain (http://mouse.brain-map.org/)^1^,^2^. Understanding the function of genes in different brain structures requires manipulation of gene expression with spatial and temporal precision. Cre recombinase, which catalyzes DNA recombination between 34-bp loxP sites^3^, is frequently used to turn gene expression on or off. Tetracycline (Tet)-dependent transactivators/repressors can temporally regulate gene expression^4^,^5^, but creating Tet responsive mouse lines typically requires significant effort and expense. Cre variants with ligand-binding domains that are only active in the presence of synthetic steroids such as RU486 or tamoxifen can also provide temporal control^6^,^7^, but invasive methods of drug delivery and toxic doses of drug may be required to obtain efficient genetic modification^8^. Specific promoters provide some degree of spatial and cellular control of gene expression^9^, but they may be imprecise, and investigators may wish to study a brain region that does not tightly match the expression of any particular promoter. Microinjection of viruses that express Cre recombinase is effective, but this produces relatively limited control over the region of expression^10^. A technique that allows permanent, precise spatiotemporal control of gene expression would vastly improve tests of gene function in specific brain regions.

Optogenetics typically involves activation of light-sensitive proteins to control a variety of biological processes, including neuronal signaling^11^. Light can also be used to induce dimerization of certain proteins, such as cryptochrome 2 (CRY2) and CIB1 from *Arabidopsis thaliana*, which interact when excited by blue light^12^,^13^. The CRY2-CIB1 system was recently used to create light-inducible transcriptional regulators that modify gene expression *in vivo*, but the genetic modification only persists when the tissue is illuminated^14^. To permanently modify gene expression using light, Kennedy *et al*. created a photo-activatable Cre recombinase (PA-Cre) via fusion of CRY2 to the N-terminal domain of Cre, and fusion of the N-terminus of CIB1 (CIBN) to the C-terminal domain of Cre (Fig. 1A)^13^. Blue light causes dimerization of CRY2 and CIBN, with consequent reconstitution of split Cre recombinase activity. PA-Cre has been used to induce expression of fluorescent proteins in *Drosophila*^15^. A different form of PA-Cre that uses a light-responsive caging group in the Cre catalytic site has been validated in mammalian cells^16^. Although these tools have enormous potential for improving spatiotemporal control of gene expression in mice, no group has shown activation of PA-Cre in a living mouse. We have now used PA-Cre to permanently modify gene expression in mouse brain with spatiotemporal precision.

## Results

### Expression and activation of PA-Cre in primary cultured neurons

**Figure 1:**
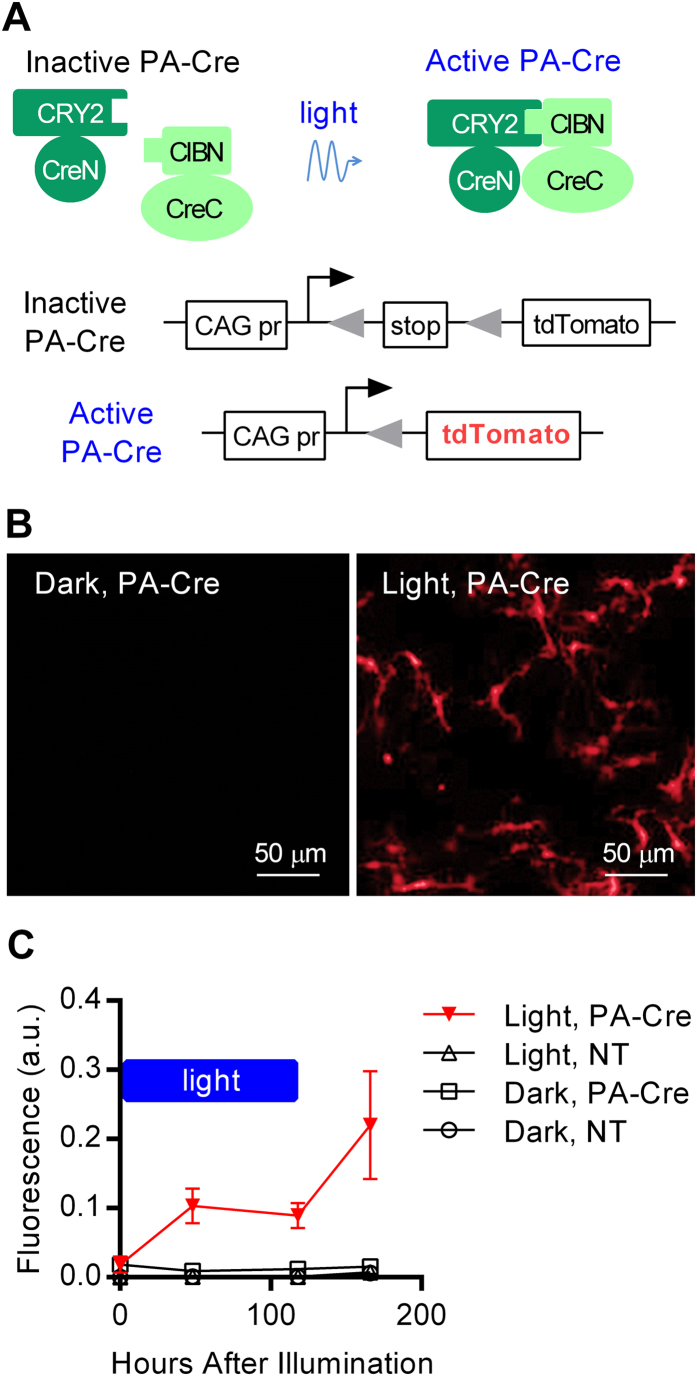
Photo-activated gene expression in primary cultured neurons. (**A**) AAV was produced to express the two components of PA-Cre: CRY2-CreN and CIBN-CreC. Blue light illumination causes dimerization of CRY2 with CIBN and reconstitution of split Cre recombinase activity. Primary cultured neurons from Ai9 mice contain a stop-floxed tdTomato gene. Cre recombinase excises the stop codon and induces expression of tdTomato. (**B**) Primary hippocampal neurons from Ai9 mice were transduced with AAV-PA-Cre on DIV5. Beginning on DIV12, neurons were illuminated with blue light pulses (466 nm x 100 ms pulse every 120 seconds) or maintained in darkness. (**C**) tdTomato expression in AAV-PA-Cre transduced cultures, as measured by flow cytometry, increased during the period of illumination (represented by the blue bar) and continued after illumination was removed. Error bars, *sd*, n = 3.

We subcloned CRY2-CreN and CIBN-CreC into Adeno-Associated Virus 2 (AAV2) plasmids, which were then packaged as AAV2/8 with the AAV8 capsid. Equal titers of AAV-CRY2-CreN and AAV-CIBN-CreC were mixed together to produce AAV-PA-Cre. To test for PA-Cre activity, we used the Ai9 Cre reporter mouse line, which contains a flox-stopped gene for tdTomato fluorescent protein inserted into the *Rosa26* locus (Fig. 1A)^17^.

We first verified that blue light activation of PA-Cre excises the floxed stop codon in cells derived from Ai9 mice, which is reported by expression of tdTomato. We transduced primary cultured neurons derived from Ai9 mice with AAV-PA-Cre at DIV5. After waiting one week to allow for PA-Cre expression, we illuminated the cells with pulsed blue light (466 nm at ~6 mW, light intensity of ~15 mW/mm^2^) or maintained them in darkness. Ai9 primary neuronal cultures transduced with AAV-PA-Cre and exposed to blue light for two or more days expressed tdTomato, indicating Cre-mediated recombination had occurred (Fig. 1B). Cells stably expressed tdTomato after illumination (Fig. 1C), indicating stable gene modification. Fewer than 0.1% of cells transduced with AAV-PA-Cre and maintained in darkness expressed tdTomato. Primary neuron cultures not transduced with AAV-PA-Cre had no cells positive for tdTomato expression.

### Activation of PA-Cre with fiber optic illumination

**Figure 2:**
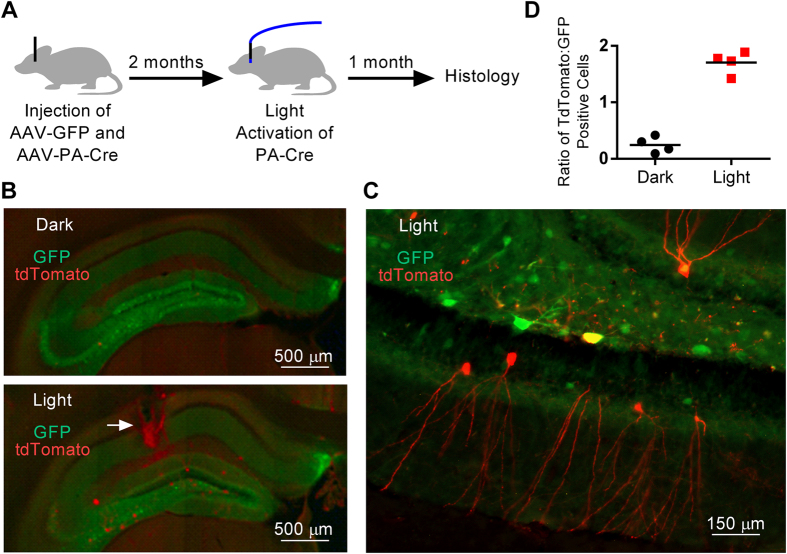
Photo-activated gene expression in the hippocampus. (**A**) The hippocampus was injected with AAV-PA-Cre and AAV-GFP. After two months, an optical fiber was implanted into the hippocampus and blue light was pulsed for 12 hours in the light but not the dark condition. One month following illumination the mice were sacrificed and brain sections were imaged. (**B**) We observed GFP expression in both the dark (top panel) and light (bottom panel) conditions. There were rare tdTomato expressing cells in the dark condition and many tdTomato expressing cells in the light condition, including cells more than 1 mm from the optical fiber (p < 0.0001 for the difference between tdTomato positive cells in light and sham conditions by Student’s T-Test). An area of gliosis surrounded the fiber optic tract (arrow) in some mice exposed to light. (**C**) Higher magnification views revealed expression of GFP and tdTomato in cells with a variety of morphologies, including cells with a typical neuronal morphology. (**D**) The ratio of tdTomato to GFP expressing cells was 7-fold higher in light-exposed animals compared to sham mice (p < 0.0001, Student’s T-Test).

We next tested whether PA-Cre could be activated in the deep structures of the brain using fiber optic illumination. We injected the hippocampus of Ai9 mice with AAV-GFP, to mark the area of transduction, and AAV-PA-Cre (Fig. 2A). AAV expression in mouse brain peaks 4–8 weeks following injection^18^, so we allowed 8 weeks for expression of PA-Cre. We then implanted an optical fiber into the hippocampus and applied blue laser light (473 nm) according to parameters previously found to initiate dimerization of CRY2 and CIB1 (5 mW, 500 ms light pulse at 0.016 Hz for 12 hours)^14^. Sham mice underwent injection of AAV-GFP plus AAV-PA-Cre and implantation of an optical fiber, but no light illumination. Transcription of genes activated by Cre recombinase can occur as early as 8–15 days following activation^7^,^19^, but to increase our chances of seeing expression, we sacrificed mice one month after illumination. We observed 4 ± 2 tdTomato positive and 18 ± 4 GFP positive cells per hippocampal slice (for example, see Fig. 2B) in the sham mice and 30 ± 4 tdTomato positive and 18 ± 2 GFP positive cells in the light activated mice (p < 0.0001 for the difference between tdTomato positive cells in light and sham conditions). Cells strongly positive for both GFP and tdTomato (~5% of tdTomato positive cells) were transduced with both AAV-PA-Cre and AAV-GFP viruses by chance. We normalized the counts of tdTomato positive cells to GFP positive cells to correct for differences in gene expression. The ratio of tdTomato to GFP expressing cells was 7-fold higher in light-exposed animals compared to sham mice (p < 0.0001) (Fig. 2D).

tdTomato positive cells had a variety of morphologies (Fig. 2C), including a typical neuronal morphology with fluorescent protein expression both in the cell bodies and neurites, reflecting the known expression of AAV8 in both neurons and glia^20^. We observed tdTomato positive cells throughout the hippocampus, indicating that blue light sufficient to trigger dimerization of CRY2 and CIB1was transmitted at least 1 mm from the optical fiber tip. This was further than expected, and suggests that sub-millimeter spatial precision in activation of PA-Cre may not be attainable with blue light at this power intensity. In 2 of the 4 mice exposed to light, a gliotic tract surrounded the optical fiber (Fig. 2B, arrow), indicating that this approach can induce tissue damage.

### Activation of PA-Cre with two-photon microscopy

Optical fibers can also be implanted in the cortex, but we hypothesized that direct illumination of the cortical surface with a microscope could activate PA-Cre with minimal tissue damage. We thus used two-photon excitation with a near-infrared laser. Compared to blue light illumination, two-photon excitation reduces light-scattering, penetrates more deeply into tissues, and is less phototoxic^21^. Two-photon stimulation at 860 nm has previously been demonstrated to induce dimerization of CRY2 and CIBN^13^.

**Figure 3:**
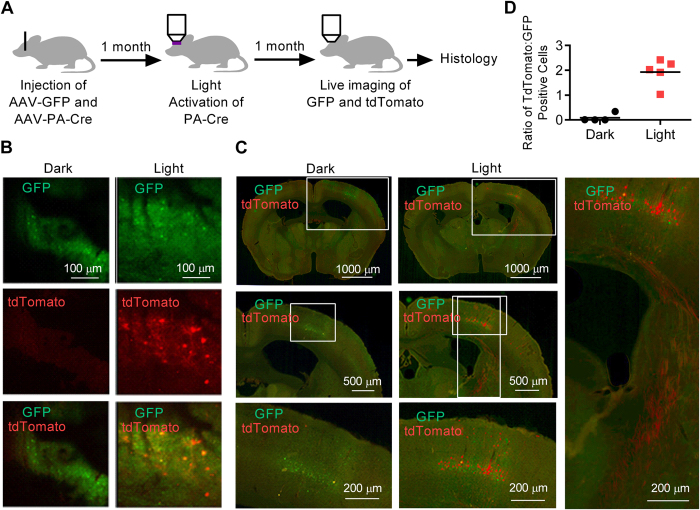
Photo-activated gene expression in the cortex of Ai9 mice. (**A**) The somatosensory cortex was injected with AAV-PA-Cre and AAV-GFP. After one month, the cortex was illuminated with an 860 nm near-infrared laser to activate PA-Cre. The cortex was scanned from the surface to a depth of 200 microns in ten 20 micron slices/frames. Scans were run in 200 cycles with an on time of 9 s (0.9 s per frame × 10 frames), and an off time of 21 s. Total scan time was ~100 minutes. (**B**) After one month the cortex was imaged in living mice. (**C**) Somatosensory cortex was imaged following sham treatment (dark) or illumination (light) at three levels of magnification. tdTomato expressing axons project from the somatosensory cortex (right panel). (**D**) The ratio of tdTomato to GFP expressing cells is represented for each mouse brain, n = 4 (dark) and n = 5 (light), p < 0.001, Student’s T-Test.

We injected AAV-PA-Cre plus AAV-GFP into the somatosensory cortex of Ai9 mice (Fig. 3A). After allowing one month for viral expression, we created a bone window by thinning the skull to transparency surrounding the injection site^22^. The region transduced by AAV-PA-Cre plus AAV-GFP was identified by the presence of GFP-expressing cells. PA-Cre was activated by imaging/scanning a 500 × 500 micron region of transduced cortex repeatedly over 9 seconds with the two-photon laser in a ten slice z-stack from the surface to a depth of 200 microns, followed by a 21 second pause for energy dispersion. We repeated this procedure for 200 cycles over 100 minutes. Sham mice had a bone window but no light exposure.

We waited one month, then imaged the cortex of living mice through the bone window. At the cortical surface, sham mice had almost zero tdTomato expression, while mice that underwent illumination expressed tdTomato in numerous cells (Fig. 3B). We sacrificed the mice and analyzed brain sections using fluorescence microscopy. We observed tdTomato expression in neuronal cell bodies within a 500 micron region, confined almost exclusively to the somatosensory cortex (Fig. 3C). Axons positive for tdTomato projected from the somatosensory cortex into the subcortical white matter tracts. In the injected region of cortex, we observed 15 ± 28 tdTomato positive and 157 ± 13 GFP positive cells in the sham mice and 281 ± 80 tdTomato positive and 146 ± 10 GFP positive cells in the light activated mice (p < 0.001 for the difference between tdTomato positive cells in light and sham conditions). Mice that underwent two-photon stimulation had a ratio of tdTomato to GFP expressing cells that was 20-fold higher than sham animals, p < 0.001 (Fig. 3D).

## Discussion

Our results demonstrate that PA-Cre can be expressed in the mouse brain and activated using illumination from either optical fibers or a two-photon microscope. Activation of PA-Cre with light occurred in minutes to hours, which is considerably faster than chemical methods that typically require drug treatment for days to weeks^4^,^5^,^6^,^7^,^8^. We waited four weeks following activation of PA-Cre to evaluate for production of tdTomato, but gene expression might be induced earlier. Compared to tamoxifen or RU486, PA-Cre will likely have few if any systemic effects. Our most exciting finding is that gene expression can be modified with sub-millimeter precision using two-photon microscopic activation of PA-Cre. This could allow very well defined regulation gene expression in discrete regions of the mouse brain. Furthermore, illumination of cortical brain structures through a thinned skull window only minimally disrupts the underlying brain structures compared to implantation of optical fibers.

Rare tdTomato expression occurred in cells not exposed to light (Figs. 2 and 3), and only in the regions transduced with AAV-PA-Cre + AAV-GFP. This reflects slightly leaky PA-Cre activity, most likely due to background binding of CRY2-CreN and CIBN-CreC. Further modification of the CRY2-CreN and CIBN-CreC constructs might optimize this tool. We also observed rare tdTomato expressing cells adjacent to the region of illumination (Fig. 3C). This may reflect activation of PA-Cre in neurites that project to cell bodies outside the region of illumination. Narrowing the zone of illumination to only the neuronal layers of the cortex may help focus gene activation on more discrete cell populations.

Transgenic expression of PA-Cre constructs with tissue-specific promoters may ultimately enable cell-type specificity (determined by the promoter) and spatial specificity (determined by the area of illumination). This approach could theoretically be translated to modify gene expression in other tissues with complex microstructures. The combination of spatial and temporal specificity provided by PA-Cre could significantly advance the study of genes within discrete brain regions.

## Methods

### Plasmid constructs

The pmCherry-CIBN-CreC and pmCherry-CRY2-CreN constructs were previously generated^13^. To create pAAV-CIBN-CreC, CIBN-CreC was amplified from pmCherry-CIBN-CreC by PCR with primers SpeI-Xhol-CIBN-F5 (CCTGTTACTAGTGAACTCGAGATGAATGGAGCTATAGGAGGTGACC) and CreC-PspOMI-R4 (CGACCGGTGGATCCCGGGCCCTAATCGCCATCTTCCAGCAGG). The PCR product was digested with SpeI+PspOMI and inserted into the AAV plasmid pTR-UF-12.1, in which the IRES-GFP was removed by digestion with SpeI+NotI.

To create pAAV-CRY2-CreN, CRY2-CreN was amplified from pmCherry-CRY2-CreN by PCR with primers SpeI- CRY2-F7-assemb (GAAGGGGTTCAAGCTTAAAAACTAGTGCCACCATGAAGATGGACAAAAAGAC) and CreN-PspOMI-R7-assem (CATGTCTGGATCCGCGCGGGCCCTTACAGCCCGGACCGACGATG). The PCR product was inserted into pTR-UF-12.1 (IRES-GFP was removed) by using Gibson Assembly Kit (New England BioLabs).

### AAV vector production

HEK293 cells were maintained in Dulbecco’s modified Eagles medium (DMEM) supplemented with 5% fetal bovine serum (FBS), 100 units/ml penicillin, 100 μg/ml streptomycin in a 37 °C incubator with 5% CO_2_. The cells were plated at 30–40% confluence in CellSTACS (Corning, Lowell, MA) 24 h before transfection (70–80% confluence when transfected). 1180 μg pHelper, 730 μg pAAV2/8 and 590 μg rAAV transfer plasmid containing the gene of interest were co-transfected into HEK293 cells using calcium phosphate precipitation. Cells were incubated at 37 °C for 72 h before harvesting. Cells were lysed by three freeze/thaw cycles. The cell lysate was treated with 50 U/ml of Benzonaze followed by iodixanol gradient centrifugation. The iodixanol gradient fraction containing the vector was further purified by column chromatography with a HiTrap Q column (GE Healthcare). The eluate was concentrated with a Vivaspin 20 100 K concentrator (Sartorius Stedim, Bohemia, NY). The viral titer was determined by dot blot.

### Mice and study approval

Ai9 mice were purchased from the Jackson Laboratory (Bar Harbor, ME) and bred at Washington University. All animal studies were performed in accordance with protocols approved by the Animal Studies Committee of Washington University.

### Photo-activated gene expression in Ai9 primary cultured neurons

The hippocampi of embryonic day 18.5 Ai9 mouse embryos were isolated and digested with 2 mg/ml papain and 0.1% DNase I. Ai9 primary cultured neurons were seeded at 60,000 cells/well onto 96-well plates pre-coated with 10 μg/ml poly-D-lysine and maintained in Neurobasal media containing B-27 and GlutaMAX. On DIV5 selected wells were transduced with PA-Cre by adding 1E10 vg of both AAV-CRY2-CreN and AAV-CIBN-CreC.

Beginning on DIV12, Ai9 primary cultured neurons in the light-exposed condition were illuminated with blue (466 nm at ~6 mW, light intensity of ~15 mW/mm^2^) light pulses of 100 ms every 120 seconds with a custom lighting set-up. At various time points following illumination, neurons were trypsinized, collected, and subjected to flow cytometry using a MACSQuant VYB (Miltenyi Biotec). Three replicates (independent wells of cells) were run for each condition. The cell suspension was excited with a 561 nm laser and cell-sized particles were gated. tdTomato fluorescence of particles was measured using a 615/20 nm band pass filter. Using FlowJo v10 software (Treestar, Ashland, OR), a threshold for tdTomato positivity was set by allowing 0.05% of non-transduced and non-illuminated cells to fall within the positive gate. The gates and thresholds were also verified using tdTomato positive Ai9 neurons transduced with wild type Cre recombinase. The fluorescence of the gated cells (in the size range of neurons) was reported.

### Fiber optic activation of PA-Cre

Three month old Ai9 mice (JAX Mice) were stereotactically injected in the right hippocampus (A/P −2.5, M/L −2.0, D/V −1.8) at an infusion rate of 0.1 μl/min with 8E9 vg each of AAV-CRY2-CreN, AAV-CIBN-CreC, and AAV-GFP in a total volume of 2 μl. After two months, an optical fiber was implanted into the hippocampus at the same coordinates. In the light-exposed condition (n = 4), a blue laser light (473 nm at ~5 mW) was pulsed at 500 ms every 60 seconds for 12 hours, following the parameters previously shown to cause dimerization of CRY2 and CIB1^14^. Mice in the sham condition (n = 4) were not exposed to laser light. One month following illumination the mice were sacrificed.

### 2-photon activation of PA-Cre

Four month old Ai9 mice (n = 10) were stereotactically injected in the right somatosensory cortex (A/P −1.0, M/L −2.5, D/V −0.7) at an infusion rate of 0.1 μl/min with 8E9 vg each of AAV-CRY2-CreN, AAV-CIBN-CreC, and AAV-GFP in a total volume of 2 μl. One month following injection, half of the mice (n = 5) underwent 2-photon microscopic activation of PA-Cre. Each mouse was anesthetized with isofluorane and the skull overlying the somatosensory cortex was thinned to transparency with a file to expose an approximately 2 mm diameter region surrounding the injection site. The anesthetized mouse was then placed on the stage of an upright Zeiss Axioskop 2 microscope coupled to a Zeiss LSM 510 Meta NLO system (Thornwood, NY). The injection site was identified based on the presence of GFP positive cells. A region of cortex (0.5 × 0.5 mm) adjacent to the injection site was activated by scanning the cortex at the thinned skull surface with a Coherent Chameleon Ultra 1 Ti:Sa laser (Santa Clara, CA) set to 860 nm at 10% power, with a measured power at the skull surface of 45 mW (light intensity of 180 mW/mm^2^). The cortex was scanned as a z-stack consisting of ten 20 micron slices starting at the surface and ending at a depth of 200 microns. Illumination of each z-stack occurred over 9 seconds (0.9 sec per frame), followed by 21 seconds for energy dispersion. A total of 200 z-stacks were acquired over ~100 minutes.

One month following 2-photon activation, the skull overlying the somatosensory cortex was removed and tdTomato expression was imaged by exciting the cortical surface at 543 nm with a helium-neon laser and collecting wavelengths >560 nm. GFP fluorescence (500–545 nm) was excited with an argon laser at 488 nm. Mice were sacrificed following imaging and histology was performed. One mouse in the dark group was excluded from analysis because no significant GFP or tdTomato expression was detected.

### Histology and quantification

Animals were perfused with ice cold 0.03% heparin in PBS followed by 4% paraformaldehyde (PFA) in PBS. Whole brains were then fixed in 4% PFA in PBS for >2 days at 4C and cryoprotected in 30% sucrose in PBS for >2 days. The brains were sectioned into 50 micron coronal slices with a freezing sliding microtome. Slices were mounted in Fluoromount G (Southern Biotech). Slides were imaged with an Olympus Nanozoomer 2.0-HT slide scanner (Hamamatsu, Bridgewater, NJ).

For optical fiber experiments, 8 hippocampal sections with GFP positive cells indicating viral expression were selected for further analysis. For 2-photon activation experiments, 6 sections through the somatosensory cortex with GFP positive were selected. tdTomato- and GFP-positive cells in each section were quantified with Visiomorph image processing software (Visiopharm, Broomfield, CO) and the counts were averaged for each mouse. The student’s two-tailed T-test was used for comparisons of tdTomato positive cells between the light and sham conditions.

## Additional Information

**How to cite this article**: Schindler, S. E. *et al*. Photo-activatable Cre recombinase regulates gene expression *in vivo*. *Sci. Rep*. **5**, 13627; doi: 10.1038/srep13627 (2015).
